# Association of Oxidative Stress Markers with Atherogenic Index of Plasma in Adult Sickle Cell Nephropathy

**DOI:** 10.1155/2012/767501

**Published:** 2012-04-18

**Authors:** M. A. Emokpae, P. O. Uadia

**Affiliations:** ^1^Department of Chemical Pathology, Aminu Kano Teaching Hospital, Kano 700001, Nigeria; ^2^Department of Medical Laboratory Science, School of Basic Medical Sciences, College of Medical Sciences, University of Benin, Benin City 300001, Nigeria; ^3^Department of Biochemistry, University of Benin, Benin City 300001, Nigeria

## Abstract

This paper evaluates the association of oxidative stress and atherogenic index of plasma in order to assess the cardiovascular risk in Sickle cell nephropathy especially as lipoprotein levels are lower in SCD than non-SCD patients. Antioxidant enzymes, malondialdehyde(MDA), urea, creatinine, and glomerular filtration rate were evaluated in 110 confirmed sickle cell disease patients: 65 males in steady state, aged 21.1 ± 6.0 years, 30 males with macroalbuminuria, aged 24.5 ± 7.0, years and 15 with chronic kidney disease (CKD), aged 31.8 ± 2.0 years. The mean activity levels of glutathione peroxidase (GPx), superoxide dismutase (Cu/ZnSOD), and catalase (CAT) were significantly lower (*P* < 0.001) in SCD with macroalbuminuria and CKD while MDA was higher (*P* < 0.001) in SCD with macroalbuminuria and CKD compared with controls. There was negative correlation between GPx (*P* < 0.001), Cu/ZnSOD (*P* < 0.02), and Atherogenic index of plasma in SCD with CKD, while MDA shows a positive correlation (*P* < 0.001) with AIP in SCD with CKD. There was however no correlation between CAT and AIP. Decreased activity levels of antioxidant enzymes and low HDL-cholesterol concentration were confirmed in adult SCD with CKD in Nigerians. The increase oxidative stress and high atherogenic index in CKD may accelerate the process of cardiovascular complications in adult SCD patients. Atherogenic index of plasma was negatively correlated with antioxidant enzymes and positively with MDA.

## 1. Introduction

 Sickle cell disease (SCD) is a haemoglobinopathy which is characterized by red blood cell rigidity, compromised perfusions, and tissue infarction [1]. The kidney of patients with SCD is affected both by haemodynamic changes of chronic and by consequences of vaso-occlusion within the renal medulla [2, 3]. Renal abnormalities in structure and function occur with increasing age of subject with SCD. The pathogenesis of SCD is due to polymerization of sickle red blood cell causing chronic haemolytic anaemia, vaso-occlusive crisis, and intravascular haemolysis. Sickle cell disease patients are susceptible to increased oxidative stress due to constant haemolysis of mutant red blood cells since haemoglobin acts as powerful catalyst for initiation of peroxidative reaction [4, 5]. Proteinuria is common in adult patients with SCD and we earlier reported a 28% prevalence of proteinuria in this group of patients in Nigeria [6]. Proteinuria is a progression factor in chronic kidney disease heralding a further deterioration in renal function [6]. Metabolic abnormalities, inflammation, and ischaemia may increase oxidative stress in sickle cell nephropathy (SCN). Increased oxidative stress in SCN due to increased pro-oxidative activity may lead to diminished antioxidant system [4, 7]. Increased oxidative stress is considered as an important pathogenic mechanism in the development of cardiovascular, cerebrovascular, and peripheral vascular complications [8, 9]. Autoperoxidation of polyunsaturated fatty acids (PUFAs) is initiated by free radicals, and the products which are oxidized in vivo to form malondialdehyde are capable of damaging membrane of biomolecules [9, 10]. Lipid abnormalities and increased oxidative stress in SCN may accelerate the process of atherosclerosis in patients with SCN. This study evaluates the association of oxidative stress and atherogenic index of plasma in order to assess the cardiovascular risk in SCN especially when lipoprotein levels are lower in SCD than non-SCD patients.

## 2. Materials and Methods

The study population was 110 confirmed SCD patients attending sickle cell disease clinic of Aminu Kano Teaching Hospital. They consisted of 65 males in steady state, aged 21.1 ± 6.0 years, 30 males with macroalbuminuria aged 24.5 ± 7.0 years, and 15 with chronic kidney disease (CKD), aged 31.8 ± 2.0 years. Demographic and clinical examination findings were obtained using structured questionnaires. The study protocol used was approved by the institute's ethical committee and the patients gave informed consent before enrolment in the study. Random urine was obtained for analysis using combi-9 commercial dipstick, which was used to test for biochemical urinalysis. Five milliliter of blood was collected aseptically and dispensed into a plain tube after 12-hour fast. The blood was allowed to clot and serum obtained after centrifugation at 3000 rpm for 10minutes. The sera were stored at −20°C and analysis was done within two weeks of collection. Urea was determined using urease colorimetric technique, creatinine was assayed using sodium hydroxide-picric acid technique, and superoxide dismutase (Cu/ZnSOD), and glutathione peroxidase (GPx) were assayed using ELISA kits supplied by Northwest life science specialities, Vancouver, Canada. Catalase was estimated using kit by SIGMA (St. Louis, Missouri, USA) and malondialdehyde was determined using thiobarbituric acid reacting substance kit supplied by Northwest life science specialties. Total cholesterol and triglyceride (TG) were determined using enzyme-catalyzed colorimetric methods by Randox laboratories, UK. HDL cholesterol was assayed using the supernatant after precipitation with magnesium chloride-phosphotungstic acid solution, while LDL cholesterol was calculated using Friedewald formula [11]. Cardiovascular risk ratio was calculated using atherogenic index of plasma (AIP) [12], which was defined as log(TG/HDL-c) with TG and HDL-c expressed in molar concentration. Glomerular filtration rate was estimated using Cockroft- Gault formular [13]. Chronic kidney disease was defined as estimated glomerular filtration rate (eGFR) of <60 mL/min and presence of macroalbuminuria. Macroalbuminuria was defined as presence in urine of albumin concentration of ≥300 mg/L. A two-sample *t*-test was used to determine the statistical significance of the means between the different groups. A *P* value of 0.05 or less was considered statistically significant. Pearson correlation coefficient was used to show the levels of association of antioxidant enzyme levels with AIP in SCD patient with CKD.

## 3. Results

 The results are as indicated in Tables 1, 2, and 3.  Table 1 shows biochemical and lipoprotein levels of SCD subjects in steady state used as controls, SCD with macroalbuminuria, and CKD. The mean serum urea and creatinine in SCD with macroalbuminuria and CKD were significantly higher (*P* < 0.001) than those of the SCD control subjects while the mean eGFR was significantly lower (*P* < 0.001) in SCD with macroalbuminuria and CKD compared with control subjects. The mean levels of triglyceride, total cholesterol, LDL cholesterol, and VLDL cholesterol were significantly higher (*P* < 0.001) in CKD compared with SCD controls while HDL cholesterol was lower (*P* < 0.001) in CKD compared with control subjects. The atherogenic index significantly increased in SCD patients with CKD compared with SCD with macroalbuminuria and control group.

Table 2 indicates changes in oxidative stress markers in the study group. The mean activity levels of GPx, Cu/ZnSOD, and CAT were significantly lower (*P* < 0.001) in SCD with macroalbuminuria and CKD while MDA was higher (*P* < 0.001) in SCD with macroalbuminuria and CKD compared with controls.

Table 3 shows the levels of association of antioxidant markers with AIP in SCD with CKD. There were negative correlations between GPx (*P* < 0.001), Cu/ZnSOD (*P* < 0.02), and AIP in SCD with CKD, while MDA shows a positive correlation (*P* < 0.001) with AIP in SCD with CKD. The correlation between CAT and AIP was however not significant.

## 4. Discussion

 The data showed that there were increases in oxidative stress and lipoprotein levels in SCD patients with macroalbuminuria and CKD. Atherogenic index of plasma was negatively correlated with antioxidant enzymes and positively with MDA in CKD. Studies have shown decreases in the activity levels of antioxidant enzymes in SCD patients in steady state [4] and in non-SCD patients with acute renal failure [14]. The present study associated the decreases in the antioxidant enzyme activity levels with AIP, used as cardiovascular risk, especially as lipoprotein levels in SCD patients are lower than non-SCD individuals in both normal and renal disease. In this study, we observed increased oxidative stress in SCD patients with CKD. Oxidative damage is due to redox imbalance between production of reactive oxygen species (ROS) and the countering effects of the various antioxidants in the body. The increased production of ROS in SCD can be grossly amplified in response to a variety of pathophysiological conditions including renal disorders, inflammation, immunologic disorders, hypoxia, metabolism of drugs or alcohol, and deficiency in antioxidant enzymes [1, 4, 15]. Reactive oxygen species can cause significant damage to biomolecules since membrane lipids readily react with ROS resulting in lipid peroxidation [16–18]. Increased oxidative stress has been reported to mediate most of the risk factors involved in kidney disease [19]. Free radical mediated injury is the primary event leading to renal injury and progressive renal insufficiency and may lead to increased levels of lipid peroxidation. This occurs because cell membrane contains large quantity of PUFAs. These PUFAs react with ROS to form peroxide derivatives [20, 21]. Oxidative damage may alter both structure and function of the glomerulus due to its effects on mesangial and endothelial cells. Overexpression of ROS by both enzymatic and nonenzymatic pathways (Fenton chemistry) promotes intravascular oxidant stress that can disrupt nitric oxide homeostasis and produce the highly oxidative peroxynitrite [22]. Previous reports have showed that renal disease in SCD has the capacity to diminish arginine bioavailability through the loss of de novo arginine synthesis from citrulline which occurs primarily in the kidneys. Renal insufficiency may impair the major route for endogenous arginine biosynthesis [23] since it plays some roles in the regulation of nitric oxide production [24]. Adults with SCD are arginine deficient even at steady state [25, 26]. 

The observed increases (*P* < 0.001) in the levels of serum triglyceride, LDL, VLDL, and decrease (*P* < 0.001) of HDL-cholesterol levels in SCD patients with CKD are consistent with other studies elsewhere and in Nigeria [27–30]. The lipid profile of SCD patients is quite lower than that in subjects with normal haemoglobin and SCD patients in Nigeria have even lower levels than SCD patients in America and the Middle East [27–29]. Several reasons have been proposed as to why there are inconsistencies between lipid studies; these include differences in age, diet, weight, smoking, gender, small sample sizes, different ranges of disease severity, and treatment regimen [31–33]. Shores et al. [28] proposed the reasons why lipoprotein levels are lower in SCD than nonSCD even in the same environmental condition to be partly due to a decrease in red blood cell volume leading to a dilution effect on plasma constituents. It may also result from strong downregulation of the synthetic pathways or downregulation of the enzymes in lipid biosynthesis. Others suggested that it is the haemolytic stress in SCD patients that is associated with a significant reduction in plasma lipids in SCD patients [29, 34].

## 5. Conclusion

Decreased activity levels of antioxidant enzymes and low HDL-cholesterol concentration were confirmed in adult SCD with CKD in Nigerians. The increase oxidative stress and high atherogenic index in CKD may accelerate the process of cardiovascular complications in adult SCD patients. Atherogenic index of plasma was negatively correlated with antioxidant enzymes and positively with MDA.

## Figures and Tables

**Table 1:** Biochemical and lipid profile of sickle cell disease controls, macroalbuminuria, and chronic kidney disease.

Variables	SCD controls	SCD macroalbuminuria	SCD CKD	Reference range
Number of subjects	65	30	15	
Age (years)	21.1 ± 6.0	24.5 ± 7.0	31.8 ± 2.0*	
Urea (mmol/L)	2.6 ± 0.9	8.3 ± 2.0*	14.2 ± 2.6*	1.7–8.3
Creatinine (*μ*mol/L)	57.3 ± 9.8	260 ± 25*	498 ± 75*	53–116
eGFR (mL/min)	101 ± 2.3	72 ± 5.0*	15.1 ± 2.0*	90–128
Triglyceride (mmol/L)	1.16 ± 0.4	1.20 ± 0.2	1.7 ± 0.25*	<1.7
Total cholesterol (mmol/L)	3.06 ± 0.5	3.45 ± 0.6*	3.9 ± 0.34*	3.1–6.2
HDL-cholesterol (mmol/L)	0.72 ± 0.2	0.70 ± 0.3	0.06 ± 0.08*	0.8–1.9
LDL-cholesterol (mmol/L)	1.90 ± 0.5	1.8 ± 0.32*	1.82 ± 0.06*	<3.99
VLDL-cholesterol (mmol/L)	0.47 ± 0.06	0.52 ± 0.06*	0.74 ± 0.06*	<0.8
AIP	0.22	0.23	0.45	<1.44
TC: HDL	4.25	4.92	6.50	<4.9
LDL: HDL	2.67	2.57	3.03	<2.4

eGFR: estimated glomerular filtration rate; AIP: atherogenic index of plasma; *P < 0.001.

**Table 2:** Oxidative stress markers of sickle cell disease control, macroalbuminuria, and chronic kidney disease.

Oxidative markers	SCD controls	SCD with macroalbuminuria	SCD with chronic kidney disease	Reference range
Number of subjects	65	30	15	
Glutathione peroxidase (mU/mL)	9.2 ± 0.9	5.54 ± 3.5*	3.01 ± 0.24*	9.2–19.6
Superoxide dismutase (ng/mL)	30.6 ± 5.1	21.3 ± 5.2*	18.5 ± 2.6*	22.5–103
Catalase (*μ*mol/min/L)	154 ± 7.9	150 ± 2.58	147 ± 1.06*	156–182
Malondialdehyde (*μ*mol/L)	2.5 ± 0.4	3.8 ± 1.7*	5.30 ± 0.3*	0.025–0.98

*P < 0.001.

**Table 3:** Correlation between antioxidant enzymes and atherogenic index of plasma in SCD with chronic kidney disease.

Correlation between parameters	*R* value	*P* value
GPx and AIP	−0.760	0.001
Cu/ZnSOD and AIP	−0.621	0.02
CAT and AIP	−0.416	NS
MDA and AIP	0.943	0.001

GPx: Glutathione peroxidase; Cu/ZnSOD: Superoxide Dismutase; CAT: Catalase and MDA: Malondialdehyde.

## References

[1] Emokpae MA, Uadia PO, Gadzama AA (2010). Correlation of oxidative stress and inflammatory markers with the severity of sickle cell nephropathy. *Annals of African Medicine*.

[2] Batinic-Haberle I, Reboucas JS, Spasojevic I (2010). Superoxide Dismutase mimics: chemistry, pharmacology and Therapeutic potential. *Antioxid Redox Signal*.

[3] Serjeant GR (1992). Renal manifestation in sickle cell disease. *Oxford Textbook of Clinical Nephrology*.

[4] Emokpae AM, Uadia PO, Kuliya-Gwarzo A (2010). Antioxidant enzymes and acute phase proteins correlate with marker of lipid peroxide in adult Nigerian sickle cell disease patients. *Iranian Journal of Basic Medical Sciences*.

[5] Klings ES, Farber HW (2001). Role of free radicals in the pathogenesis of acute chest syndrome in sickle cell disease. *Respiratory Research*.

[6] Abdu A, Emokpae M, Uadia P, Kuliya-Gwarzo A (2011). Proteinuria among adult sickle cell anemia patients in Nigeria. *Annals of African Medicine*.

[7] Steinberg MH (2006). Pathophysiologically based drug treatment of sickle cell disease. *Trends in Pharmacological Sciences*.

[8] Hebbel RP, Eaton JW, Balasingam M, Steinberg MH (1982). Spontaneous oxygen radical generation by sickle erythrocytes. *Journal of Clinical Investigation*.

[9] Yam J, Frank L, Roberts RJ (1978). Oxygen toxicity: comparison of lung biochemical responses in neonatal and adult rats. *Pediatric Research*.

[10] Fridovich I, Freeman B (1986). Antioxidant defenses in the lung. *Annual Review of Physiology*.

[11] Friedewald WT, Levy RI, Fredrickson DS (1972). Estimation of the concentration of low-density lipoprotein cholesterol in plasma, without use of the preparative ultracentrifuge. *Clinical Chemistry*.

[12] Cockcroft DW, Gault MH (1976). Prediction of creatinine clearance from serum creatinine. *Nephron*.

[13] Manfredini V, Lazzaretti LL, Griebeler IH, Santin AP, Brandao VD, Wagner S (2008). Blood antioxidant parameters in sickle cell anaemia patients in steady state. *National Medical Association*.

[14] Ramani G, Kavitha G, Dhass PK, Aruna RM (2011). Oxidative stress and its association with cardiovascular risk in acute renal failure. *International Journal of Pharma and Bio Sciences*.

[15] Remuzzi G, Bertani T (1998). Pathophysiology of progressive nephropathies. *New England Journal of Medicine*.

[16] Pawlak K, Pawlek D, Mysliwice M (2005). Cu/Zn superoxide dismutase plasma level as a new useful chemical biomarker of oxidative stress in patients with end stage renal disease. *Clinical Biochemistry*.

[17] Arinola OG, Olaniyi JA, Akiibinu MO (2008). Evaluation of antioxidant levels and trace element status in Nigerian sickle cell disease patients with Plasmodium parasitaemia. *Pakistan Journal of Nutrition*.

[18] Sutton TA, Mang HE, Campos SB, Sandol RM, Yoder MC, Molitoris BA (2003). Injury of the renal microvascular endothelium alters banner function after ischemia. *American Journal of Physiology*.

[19] Ratych RE, Bulkley GB (1986). Free-radical-mediated postischemic reperfusion injury in the kidney. *Journal of Free Radicals in Biology and Medicine*.

[20] Mark SP, John RH, Thomas FF (1984). Oxygen free radicals in Ischaemic acute renal failure in the rat. *Journal of Clinical Investigation*.

[21] Greene EL, Paller MS (1991). Oxygen free radicals in acute renal failure. *Mineral and Electrolyte Metabolism*.

[22] Emokpae MA, Uwumarongie OH, Osadolor HB (2011). Sex dimorphism in serum lecithin: cholesterol acyltransferase and lipoprotein lipase activities in adult sickle cell anaemia patients with proteinuria. *Indian Journal of Clinical Biochemistry*.

[23] Wood KC, Hsu LL, Gladwin MT (2008). Sickle cell disease vasculopathy: a state of nitric oxide resistance. *Free Radical Biology and Medicine*.

[24] Morris CR (2008). Mechanisms of vasculopathy in sickle cell disease and Thalassemia. Current and Future Therapies of sickle cell anemia. *Hematology*.

[25] Morris CR, Teehanke C, Kato G Decreased arginine bioavailability contributes to the pathogenesis of pulmonary artery hypertension.

[26] Enwonwu CO (1989). Increased metabolic demand for arginine in sickle cell anaemia. *Medical Science Research*.

[27] Emokpae MA, Abdu A, Uadia PO, Borodo MM (2010). Lipid profile in sickle cell disease patients with chronic kidney disease. *Sahel Medical Journal*.

[28] Shores J, Peterson J, VanderJagt D, Glew RH (2003). Reduced cholesterol levels in African-American adults with sickle cell disease. *Journal of the National Medical Association*.

[29] Rahimi Z, Merat A, Haghshenass M, Madani H, Rezaei M, Nagel RL (2006). Plasma lipids in Iranians with sickle cell disease: hypocholesterolemia in sickle cell anemia and increase of HDL-cholesterol in sickle cell trait. *Clinica Chimica Acta*.

[30] Morris CR, Kuypers FA, Larkin S, Vichinsky EP, Styles LA (2000). Patterns of arginine and nitric oxide in patients with sickle cell disease with vaso-occlusive crisis and acute chest syndrome. *Journal of Pediatric Hematology/Oncology*.

[31] Zailaie MZ, Marzouki ZM, Khoja SM (2003). Plasma and red blood cells membrane lipid concentration of sickle cell disease patients. *Saudi Medical Journal*.

[32] Choy E, Sattar N (2009). Interpreting lipid levels in the context of high-grade inflammatory states with a focus on rheumatoid arthritis: a challenge to conventional cardiovascular risk actions. *Annals of the Rheumatic Diseases*.

[33] Gotto APH (2003). *Manual of Lipid Disorders: Reducing the Risk for Coronary Heart Disease*.

[34] Zorca S, Freeman L, Hildesheim M (2010). Lipid levels in sickle-cell disease associated with haemolytic severity, vascular dysfunction and pulmonary hypertension. *British Journal of Haematology*.

